# Position paper by the UKCCCR Elderly Cancer Patients in Clinical Trials Working Group

**DOI:** 10.1054/bjoc.1999.0866

**Published:** 1999-12-08


*British Journal of Cancer* (2000) **82**(1), 1–3
© 2000 Cancer Research Campaign
Article no. bjoc.1999.0866

### Position paper by the UKCCCR Elderly Cancer Patients in Clinical Trials Working Group

### BACKGROUND

Following discussions at the UKCCCR Trials Committee in 1995, a decision was taken during early 1996 to set up a Working Group to consider the management of elderly cancer patients and their entry into clinical trials. This decision was subsequently endorsed by the Main Committee of the UKCCCR. Dr Peter Harper (Guy's Hospital, London) kindly agreed to chair the Group and the other members were then recruited (see below). The Group met for the first time in July 1996 and on five further occasions up until October 1997. This short paper summarizes the proceedings to date.

### TERMS OF REFERENCE

The following Terms of Reference for the Group were drawn up by Dr Peter Twentyman (Executive Secretary, UKCCCR) and Professor Nick Thatcher (Chairman, UKCCCR Trials Committee). These were agreed and adopted by the first meeting of the Group.

To advise the UKCCCR upon:

a. why relatively few elderly patients are entered into cancer clinical trials and whether any action could help the situation.
b. whether research (including clinical trials) specifically aimed at treatment of elderly patients is needed.
c. any other related questions which the Working Group consider appropriate.

### THE PROBLEM

Cancer is predominantly a disease of the elderly. With the current trend in the UK towards an ageing population, treatment of cancer in the elderly will become a greater problem. By 1996, 91% of male and 89% of female deaths from malignant neoplasia occurred in the 55+ age group, whilst 42% and 48% respectively were deaths in men and women aged 75 and over (Office of National Statistics, 1997). Randomized controlled clinical trials are currently considered the 'gold standard' for the development of new treatments for cancer. However, there is evidence that relatively few elderly patients are entered into such trials (Tremble et al, 1994). In a recent South West Oncology Group Study in the USA, it was found that the elderly represented only 25% of the 15 500 total participants despite comprising 63% of the cancer patients population (Unger et al, 1998). Hence, elderly patients are deprived of the potential benefit of 'state of the art' treatments and the age-spectrum upon which a new treatment is tested does not reflect that of the general population of patients who will receive the new treatment if it becomes standard. In the USA, positive initiatives to address these issues have been taken (Castellucci, 1999).

There is substantial evidence to indicate that, with a range of tumour types, given similar treatments, elderly patients have a relative survival similar to that of younger patients (Begg and Carbone, 1983; Dhodapkar et al, 1996; Siu et al, 1996). However, a number of studies have shown that elderly patients often have more advanced tumours at the time of diagnosis (Bergman et al, 1992; Busch et al, 1996; Goodwin et al, 1996) and receive less aggressive treatment (Newcomb and Carbone, 1993; August et al, 1994; Higtower et al, 1994; McKenna, 1994; Newschaffer et al, 1996) than their younger counterparts. Although most of these reports emanate from North America, it seems likely that a similar situation exists in the UK. Certainly a number of organizations in the UK (e.g. Age Concern England) are concerned that elderly patients frequently receive inadequate treatment. It is worth noting that the Health of the Nation mortality target for breast cancer refers only to 'the population invited for screening' (i.e. those under 65) (Department of Health, 1995) and it is likely that newer targets will also exclude older women.

**Membership**

The present membership of the Group is as follows:

| | |
|---|---|
| Dr Peter Blake | The Royal Marsden Hospital, London |
| Professor J Grimley Evans | The Radcliffe Infirmary, Oxford |
| Professor Lesley Fallowfield | CRC Psychosocial Oncology Group, UCLMS |
| Professor Ian Fentiman | Guy's Hospital, London |
| Dr Margot Gosney | Royal Liverpool Hospital, Liverpool |
| Dr Emily Grundy | Centre for Population Studies, London School of Hygiene & Tropical Medicine |
| Dr Peter Harper | Guy's Hospital, London (Chairman) |
| Ms Hazel Heath | Chair, RCN Forum for Nurses Working with Older People |
| Dr Iona Heath | The Kentish Town Health Centre, London |
| Professor Kay-Tee Khaw | Addenbrooke's Hospital, Cambridge |
| Mr John Northover | St Mark's Hospital, Middlesex |
| Ms Gill Oliver | Clatterbridge Centre for Oncology, The Wirral |
| Ms Val Speechley | Royal Marsden Hospital, London |
| Dr Peter Twentyman | UKCCCR, London (Secretary) |
| Ms Jane Whelan | Age Concern England, London |

### REPRESENTATION OF ELDERLY PATIENTS IN TRIALS

Clearly there are different opinions as to the age at which a patient can reasonably be regarded as 'elderly'. For the present purposes, however, a cut-off at 65 appears to be an acceptable working basis.

In the past, many cancer clinical trials have specifically listed age limits within the inclusion/exclusion criteria. The practice has, however, recently become much less common. Whilst it is widely believed that elderly patients are currently under-represented in trials without such obvious age limits, there do not appear to be any UK data available to substantiate this belief. In general, UK Cancer Registries do not include, within their standard data set, whether or not patients are entered into trials (personal communication to P Twentyman). However, the Thames Cancer Registry are intending to include this information in future (personal communication to P Twentyman). Information regarding the age distribution of patients being diagnosed or dying of specific cancer types is available from cancer registries. Furthermore, data are stored by individual cancer trials offices regarding the age distribution of patients being entered into trials. Hence comparison of such data sets may potentially give an appropriate guide to the extent of the problem. A more definitive approach, however, would require the collation and comparison of data sets based on the same population. This would be a quite difficult exercise which would need specific funding as a research exercise to be carried out jointly by cancer registry/trials office personnel.

### REASONS FOR POOR ENTRY

Within the Group, it is believed that a number of possible reasons could explain the poor representation of elderly patients in trials.

These could include:

a. Previous explicit upper age limits
b. Late presentation by patient
c. GP's decision not to refer the patient for active therapy
d. Consultant's decision not to offer active therapy
e. Consultant's decision not to offer trial entry
f. Patient's refusal to accept randomization.

A definitive study of the relative importance of these reasons can probably not be based on self-completion questionnaires sent to patients, GPs or consultants. These would be likely to have a low rate of return and be regarded as over-simplistic. Similarly, examination of hospital notes or other records is unlikely to be particularly helpful on its own. A worthwhile study would require structured interviews with patients/doctors, carried out by experts in qualitative psychosocial research (Meredith, 1996). This would require significant funding.

There is belief within the Group that elderly patients often have different expectations of doctors than younger patients and were much more likely to rely unquestioningly on the doctor's opinion rather than making their own decision. It is a matter of continuing debate as to whether a patient who clearly states (in an ethical and non-persuasive situation) a wish that the doctor makes all the decisions can be regarded as having given informed consent to trial entry (Tobias, 1997).

It remains, of course, unproven that under-representation of elderly patients into trials has, in itself, a detrimental effect on their treatment. However, in the light of evidence that, in general, patients entered into clinical trials (even those in control arms) do better than patients not entering trials (Davis et al, 1985; Karjalainen and Palva, 1989), this would seem very likely to be the case. More generally, under-representation in trials is likely to be detrimental to the development of optimal care for older patients as a group.

### SPECIFIC PROBLEM – TOXICITY OF TREATMENT

The Group considered the general question of whether the toxicity of treatment was likely to be greater in elderly patients. There was no strong feeling that this was the case, and certainly, the published evidence does not generally support this view (Begg and Carbone, 1983; Giovanazzi-Baannon et al, 1994; Monfardini et al, 1995). Indeed some of the newer chemotherapuetic agents appear to have a particular beneficial therapeutic index in elderly patients (Lichtman, 1998). However, co-morbidity can prevent the administration of some potentially beneficial interventions (Satariano, 1993; Newschaffer et al, 1996). It was agreed that some clinicians may be reluctant to enter elderly patients as they felt that failure to complete the prescribed treatment without dose reduction was more likely in such patients. The Group took the view, however, that trial protocols should usually include provision for dose reduction in a way which did not bias entry against patients for whom dose reduction was perceived as more likely.

### SPECIFIC PROBLEM – QUALITY OF LIFE DATA

Quality of life measures are now an important aspect of clinical trials of new cancer therapies. There is evidence that elderly people may rate their quality of life more highly than the proxy assessments made of them by their carers (Baur and Okun, 1983; Ganz, 1993) and, also, that they regard quality of life issues as a higher priority in clinical decision-making than younger patients (McKenna, 1994; Yellen et al, 1994). Furthermore, elderly patients' quality of life scores may be superior to those found in younger people (Yellen et al, 1994). There are several reasons for these findings including the fact that elderly patients may indeed be less functionally and psychologically impaired than stereotypical expectations would suggest (Yellen et al, 1994). Elderly people may also make comparative judgements, rating themselves as performing well 'for someone of my age and stage' (Grimley Evans, 1992). Quality of life assessment in older people is obviously an area worthy of considerably more research especially as cancer is predominantly a disease of the elderly.

### CONCLUSIONS

Given the current state of knowledge, the Group would recommend that:

1. Age, per se, should not be an exclusion factor for clinical trials. Criteria such as performance status, creatinine clearance, etc. (which may show correlation with age) should be used instead.
2. Clinical trials directed specifically at the elderly should only take place where there is clear evidence that the biology of the target disease is different in the elderly (e.g. AML). (However, it could be argued that inclusion should still be determined by the biological parameters rather than age per se.)

© 2000 Cancer Research Campaign

3. Trialists should be more pragmatic when designing trials, aiming to list inclusion criteria rather than exclusion criteria.
4. For trials where not all patients are entered into quality of life and/or health economic studies, there should be separate sections investigating these for the elderly patients who are recruited.
5. Clinicians should not be encouraged to 'pressurize' elderly patients into entering trials. Ideally, the proportion of elderly patients offered trial entry should be similar to that for all patients, although the number actively accepting may be lower.
6. It may be that, in future, clinical trials of palliative/terminal care should be given a higher priority. This would be likely to shift trial resources towards a more elderly population and may spread trial benefit more evenly amongst the cancer patient population.
7. There is a need for substantial further funded research into problems associated with the entry of elderly patients into trials. Specific areas of research should include:

   a. Investigation of the reasons for the late presentation by elderly patients
   b. Identification of cases of late diagnosis in general practice and strategies for improvement
   c. Examination of GP attitudes towards cancer in the elderly
   d. Detailed studies of temporal changes, and the underlying reasons, in representation of older patients in clinical trials
   e. Detailed studies of outcomes of cancer treatment in relation to patient age, and temporal changes in these.
   f. Clinical trials aimed at determining optimal treatments for frail cancer patients, irrespective of age.

Such research may help to ensure that the distribution of ages of patients entering trials more accurately reflects the age distribution of patients who will receive the novel treatment if it enters into routine procedure. (This is important not only for a survival end point but also for quality of life and/or health economic measures.) In the longer term, this should improve the treatment of cancer in the elderly by ensuring that a larger population of elderly patients are offered the most modern therapies.

### REFERENCES

August DA, Rea T and Sondak VK (1994) Age-related difference in breast cancer treatment. *Ann Surg Oncol* **1**: 45–52

Baur P and Okun MA (1983) Stability of life satisfaction in late life. *Gerontologist* **23**: 261–265

Begg CB and Carbone PP (1983) Clinical trials and drug toxicity in the elderly. The experience of the Eastern Cooperative Oncology Group. *Cancer* **52**: 1986–1992

Bergman L, Kluck HM, van Leeuween FE, Crommelin MA, Dekker G, Hart AA and Coebergh JW (1992) The influence of age on treatment choice and survival of elderly breast cancer patients in south-eastern Netherlands: a population-based study. *Eur J Cancer* **28a**: 1475–1480

Browne JP, O'Boyle CA, McGee HM, Joyce CR, McDonald NJ, O'Maley K and Hiltbrunner B (1994) Individual quality of life in the healthy elderly. *Qual Life Res* **3**: 235–244

Busch E, Kemeny M, Fremgen A, Osteen RT, Winchester DP and Clive RE (1996) Patterns of breast cancer care in the elderly. *Cancer* **78**: 101–111

Castellucci L (1999) Better fundamentals – not 'razzle dazzle' – needed in cancer research on the elderly. *J Natl Cancer Inst* **91**: 14–16

Davis S, Wright PW, Schulman SF, Hill LD, Pinkham RD, Johnson LP, Jones TW, Kellogg HB, Radke HM, Sikkema WW, Jolly PC and Hammar SP (1985) Participants in prospective, randomized clinical trials for resected non-small cell lung cancer have improved survival compared with nonparticipants in such trials. *Cancer* **56**: 1710–1718

Department of Health (1995) The Health of the Nation. Fit for the future: second progress report on the health of the nation. DOH: London

Dhodapkar MV, Ingle JN, Cha SS, Mailliard JA and Wieand HS (1996) Prognostic factors in elderly women with metastatic breast cancer treated with tamoxifen: an analysis of patients entered on four prospective clinical trials. *Cancer* **77**: 683–690

Ganz PA (1993) Age and gender as factors in cancer therapy. *Clin Geriatr Med* **9**: 145–155

Giovanazzi-Baannon S, Rademaker A, Lai G and Benson AB III (1994) Treatment tolerance of elderly cancer patients entered onto phase II clinical trials: an Illinois Cancer Center study. *J Clin Oncol* **12**: 2447–2452

Goodwin JS, Samet JM and Hurt WC (1996) Determinants of survival in older cancer patients. *J Natl Cancer Inst* **88**: 1031–1037

Grimley Evans J (1992) Quality of life assessments and elderly people. In: *Measures of the Quality of Life*, Hopkins A (ed), pp. 107–116. Royal College of Physicians: London

Higtower RD, Nguyen HN, Averette HE, Hoskins W, Harrison T and Steren A (1994) National survey of ovarian carcinoma. IV: Patterns of care and related survival for older patients. *Cancer* **73**: 377–383

Karjalainen S and Palva I (1989) Do treatment protocols improve end results? A study of survival of patients with multiple myeloma in Finland. *Br Med J* **299**: 1069–1072

Lichtman S (1998) Recent developments in the pharmacology of anticancer drugs in the elderly. *Current Opinion in Oncology* **10**: 572–579.

Meredith C, Symonds P, Webster L, Lamont D, Pyper E, Gillis CR and Fallowfield L (1996) Information needs of cancer patients in west Scotland: cross sectional survey of patients' views. *Br Med J* **313**: 724–726

McKenna RJ Sr (1994) Clinical aspects of cancer in the elderly. Treatment decisions, treatment choices, and follow-up. *Cancer* **74**: 2107–2117

Monfardini S, Sorio R, Boes GH, Kaye S and Serraino D (1995) Entry and evaluation of elderly patients in European Organization for Research and Treatment of Cancer (EORTC) new-drug-development studies. *Cancer* **76**: 333–338.

Newcomb PA and Carbone PP (1993) Cancer treatment and age: patient perspectives. *J Natl Inst* **85**: 1580–1584.

Newschaffer CJ, Penberthy L, Desch CE, Retchin SM and Whittemore M (1996) The effect of age and comorbidity in the treatment of elderly women with nonmetastatic breast cancer. *Arch Intern Med* **156**: 85–90.

Office of National Statistics (1997) Deaths registered in 1996 by cause and by area of residence. ONS Population and Health Monitor DH2 97.

Satariano WA (1993) Ageing, comorbidity and breast cancer survival: an epidemiologic view. *Adv Exp Med Biol* **330**: 1–11.

Siu LL, Shepherd FA, Murray N, Feld R, Pater J and Zee B (1996) Influence of age on the treatment of limited-stage small-cell lung cancer. *J Clin Oncol* **14**: 821–828

Tobias JS (1997) BMJ's present policy (sometimes approving research in which patients have not fully given informed consent) is wholly correct. *Br Med J* **314**: 1111–1114.

Tremble EL, Carter CL, Cain D, Freidlin B, Ungerleider RS and Friedman MA (1994) Representation of older patients in cancer treatment trials. *Cancer* **74**: 2208–2214.

Unger J, Hutchins J, Crowley C, Coltman C and Albain K (1998) Southwest Oncology Group (SWOG) accrual by sex, race and age, compared to US population rates. ASCO Proceedings, abstract 1596.

Yellen SB, Cella DF and Leslie WT (1994). Age and clinical decision making in oncology patients. *J Natl Cancer Inst* **86**: 1766–177

*© 2000 Cancer Research Campaign*
